# Diet-Induced Obesity Alters Granulosa Cell Transcriptome and Ovarian Immune Environment in Mice

**DOI:** 10.3390/life15030330

**Published:** 2025-02-20

**Authors:** Minseo Lee, Sujin Son, Surim Oh, Eunbin Shin, Hyejin Shin, Ohrim Kwon, Sohyun Hwang, Haengseok Song, Hyunjung Jade Lim

**Affiliations:** 1 Department of Veterinary Medicine, School of Veterinary Medicine, Konkuk University, Seoul 05029, Republic of Korea; 2 Korean Medicine Convergence Research Division, Korea Institute of Oriental Medicine, Daejeon 34054, Republic of Korea; 3 Department of Life Science, Graduate School, CHA University, Seongnam-si 13488, Gyeonggi-do, Republic of Korea; 4 Department of Pathology, CHA Bundang Medical Center, CHA University School of Medicine, Seongnam-si 13520, Gyeonggi-do, Republic of Korea; 5 Department of Biomedical Science and Technology, Institute of Biomedical Science & Technology, Konkuk University, Seoul 05029, Republic of Korea

**Keywords:** granulosa cell, obesity, mouse, ovary, *Egr1*

## Abstract

Obesity affects female reproductive performance by impairing the ovarian and uterine environments. Using a diet-induced obesity mouse model, we examined whether a high-fat diet (HFD) regimen affects the gene expression profile in ovarian granulosa cells (GCs) and whether short-term HFD has similar effects on gene expression as long-term HFD. C57BL/6J mice were fed a HFD or normal diet (ND) for 16–18 weeks (long-term group) or 4 weeks (short-term group). GCs were collected from each group of mice for RNA-sequencing. RT-PCR and immunofluorescence staining were performed to validate the results. RNA-sequencing analyses of the GCs revealed that several immediate early genes, including *early growth response 1* (*Egr1*), an important mediator of ovulation, were significantly downregulated in HFD GCs. *Protein tyrosine phosphatase receptor type C* (*Ptprc*) and *hematopoietic type prostaglandin D synthase* (*Hpgds*), both of which are associated with increased inflammation, were significantly upregulated in HFD GCs. Downregulation of *Egr1* was also confirmed in the GCs of short-term HFD mice, suggesting that it constitutes an early change in response to a HFD. Increased expression of several transcription factors in HFD GCs suggests that a HFD may affect the overall transcriptional landscape. The results may indicate possible modulation of the immune environment in HFD ovaries. These results provide novel insights into the molecular changes in GCs in obese environments.

## 1. Introduction

Diverse factors are held responsible for the worldwide increase in obesity rates in modern societies. Changes in dietary habits and reduced physical activity during the COVID-19 pandemic have also contributed to the recent rise in obesity among young people [1]. In women, obesity negatively affects fertility through complex mechanisms and is closely associated with an increased risk of many female reproductive conditions and fertility [2,3,4]. Polycystic ovary syndrome, reduced ovulation, and poor oocyte quality have been reported in women with obesity and in rodent models fed a high-fat diet (HFD) [5,6,7]. Uterine bleeding, endometriosis, preeclampsia, and miscarriage also increase in obese individuals [8]. Although the precise mechanisms underlying these issues have not been firmly established, it is evident that the ovarian environment adversely affects the reproductive health of women with obesity [4]. Owing to the aforementioned adverse effects of obesity on the reproductive health of women, the proportion of women with obesity seeking medical intervention to become pregnant is higher than that of women with normal weight [9].

Studies on the effects of obesity on reproductive health have widely used rodent models fed HFD chow for specific periods [10,11]. The C57BL/6J mouse strain is widely used to investigate mechanisms underlying HFD-induced physiological and cellular changes in several systems [12]. When mice are fed HFD chow with a 60% fat content, they exhibit a range of physiological changes that mimic those occurring in obese humans [3,11,13]. A shorter period of HFD (~4 weeks) was shown to induce hyperglycemia and hyperinsulinemia in DIO mice, and prolonged HFD (~4 months) is accompanied by visceral fat accumulation, increased total fat mass, diabetes, and hypertension [12]. Prolonged HFD exposure (4 months) has been shown to impair ovulation, delay preimplantation embryo development, and cause post-implantation fetal growth defects [14], but whether short-term HFD influences the aspects of reproduction is not clear. Under HFD conditions, various organs and cells show changes in intracellular signaling pathways and increased apoptotic cell death [15,16]. Such molecular and cellular changes are collectively considered lipotoxic effects, as they result from excessive accumulation of fatty acids or lipid metabolites [15]. In various studies, HFD-fed mice showed obesity-induced changes in oocyte and follicular environments [7,17,18,19,20,21].

Granulosa cells (GCs) surround growing oocytes within ovarian follicles and maintain close cell–cell communication during oocyte growth and ovulation [22]. These cells communicate with oocytes during oocyte development, follicle growth, ovulation, and hormonal regulation [23]. Therefore, exploring how GCs respond to a HFD environment is important to understand the changes that occur in oocytes and developing embryos after fertilization [24,25]. In this study, we investigated the changes in gene expression profiles in the GCs of HFD-fed mice to understand their effect on the ovarian environment. We report that *early growth response 1* (*Egr1*) and other immediate early genes encoding transcription factors are suppressed in HFD GCs. We also found that immune cell-associated factors are affected by a HFD in GCs, providing novel insights into the molecular changes in the GC environment in obesity.

## 2. Materials and Methods

### 2.1. Animals and Diet

**Figure 1:**
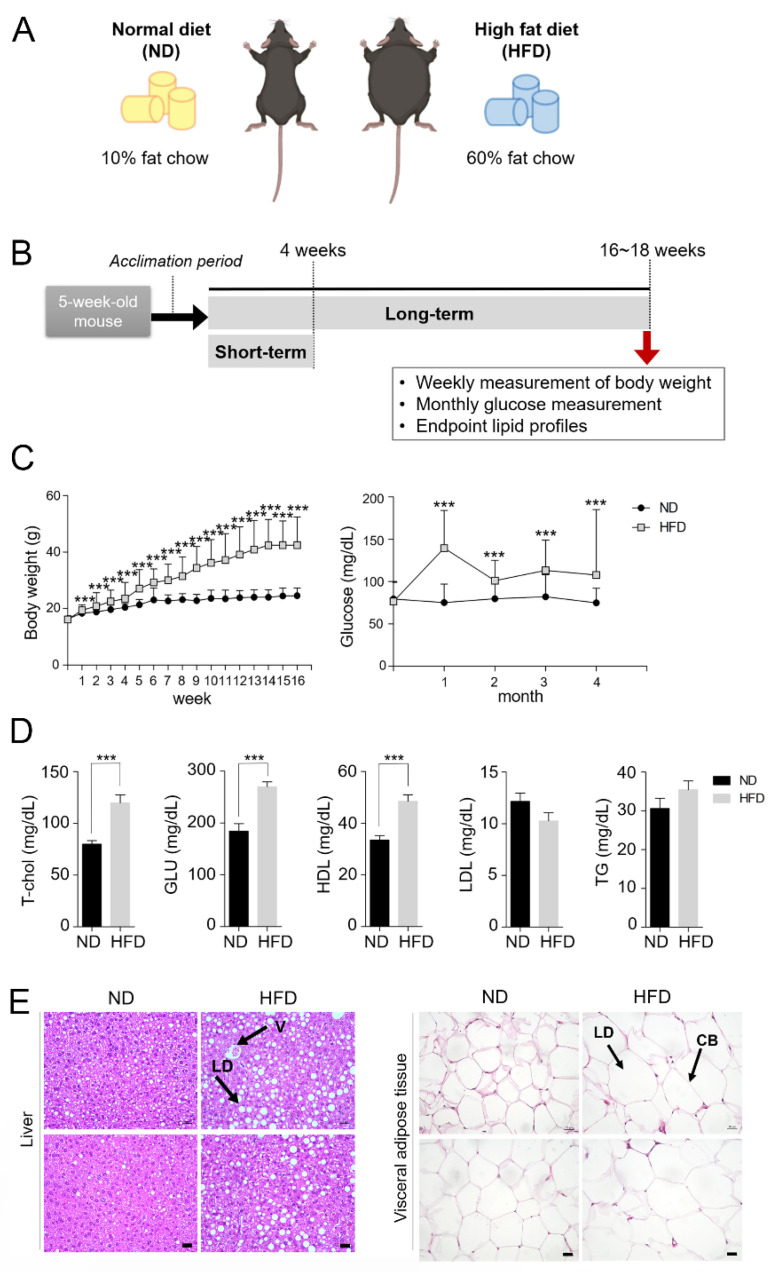
Serological and histological features of diet-induced obesity mice. (**A**) A schematic of the two groups of experimental C57BL/6J mice used in the present study. Normal diet (ND) mice were fed 10% fat chow, and high-fat diet (HFD) mice were fed 60% fat chow. (**B**) “Short-term” groups received a HFD for four weeks, whereas the “long-term” groups received a HFD for 16–18 weeks. Blood was drawn from the posterior vena cava of the mice at the experimental endpoint, and their lipid profiles were examined. (**C**) Body weight and fasting glucose levels were monitored at the indicated intervals. The body weights of mice in the ND and HFD groups were measured weekly starting at the beginning of the experiment (week 0, left panel). The fasting glucose level in blood obtained from the tip of the tail was measured once monthly (right panel). (**D**) The levels of total cholesterol (T-chol), glucose (GLU), high-density lipoprotein (HDL), low-density lipoprotein (LDL), and triglyceride (TG) were measured at the experimental endpoint. Twenty-five mice per group were used for these measurements. Statistical analysis was performed using the *t*-test. *** *p* < 0.001. (**E**) Histological analysis of the liver and visceral adipose tissues from ND and HFD mice. Tissues were collected at the experimental endpoint and stained with hematoxylin–eosin. Representative images of hematoxylin–eosin-stained paraffin sections of the liver and visceral adipose tissue showing enlarged lipid droplets in the HFD group (ND mice [*n* = 3], HFD mice [*n* = 3]). LD, lipid droplet; V, blood vessel; CB, cell boundary. Scale bar = 10 µm.

All the mice were maintained according to the guidelines of the International Animal Care and Use Committee (IACUC) of Konkuk University. The study was approved by Konkuk University IACUC (KU22157; approved on 22 Aug 2022 and KU23193; approved on 11 September 2023). Five-week-old female C57BL/6J mice (*n* = 100) were purchased from Raon Bio (Yongin-si, Republic of Korea). At the beginning of the experiments, the mice were fed regular chow for one week and then randomly divided into two groups. One group (*n* = 25) was fed a normal diet containing 10% fat (ND, D12450B, Research Diets, New Brunswick, NJ, USA), and the other group (*n* = 25) was fed a HFD containing 60% fat (HFD, D12492, Research Diets). The special chow feeding was initiated at 6 weeks of age and continued for 14–16 weeks in the long-term groups or for 4 weeks in the short-term groups (Figure 1A,B).

### 2.2. Collection of Blood and Serum Analyses

To collect blood samples for biochemical analyses, the mice were fasted for 12 h. Before drawing blood from the posterior vena cava the following morning, they were euthanized with Avertin (2,2,2-tribromoethanol in PBS, 250 mg/kg body weight, intraperitoneal injection). The first injection was given to put mice to sleep, and then, the second dose was given for euthanasia. During this process, all efforts were made to minimize suffering. Blood was drawn from the posterior vena cava and placed in a serum-separating tube. The blood samples were incubated for 30 min at 25 °C and then centrifuged at 850× *g* for 10 min at 25 °C. The supernatants were subjected to serum lipid analysis (total cholesterol [T-chol], glucose [GLU], triglyceride [TG], high-density lipoprotein [HDL], and low-density lipoprotein [LDL]). Data analysis was outsourced to the Korea Non-Clinical Technology Solution Center (Seongnam-si, Republic of Korea).

### 2.3. Measurement of the Body Weight and Fasting Glucose Levels

The body weights of all the animals were monitored weekly. To measure the fasting glucose levels, mice were fasted for 12 h before the test. A drop of blood was drawn from the tip of the tail, and the glucose level was monitored every four weeks using a glucometer.

### 2.4. Histological Analysis of Liver and Visceral Adipose Tissue

Liver and visceral adipose tissues were isolated from ND and HFD mice and fixed in 4% paraformaldehyde (PFA; Biosesang, Yongin-si, Republic of Korea) overnight. The tissues were then washed in PBS, dehydrated in serial dilutions of ethanol, and cleared in xylene. The tissues were embedded in paraffin, and sections were cut at a 10 μm thickness. The tissue sections were stained with hematoxylin–eosin (H&E) (Korea Non-Clinical Technology Solution Center). Slides were examined with an upright microscope (Nikon, Tokyo, Japan).

### 2.5. Isolation of Ovarian GCs

Ovarian GCs were isolated according to a protocol described by Campbell et al. [26]. Following the designated period of feeding, the ND or HFD mice were intraperitoneally injected with 10 IU of pregnant mare serum gonadotropin (PMSG; Daesung Microbiological Labs, Uiwang-si, Republic of Korea). The ovaries were isolated 48 h post-injection. To prepare the GCs, ovaries from four mice were pooled and subjected to GC isolation. Briefly, isolated ovaries were treated with 0.5 M ethylene glycol-bis-N,N,N′,N′-tetraacetic acid (EGTA; AG Scientific, San Diego, CA, USA) for 15 min, followed by incubation in hypertonic sucrose solution containing 0.5 M sucrose (Thermo Fisher Scientific, Waltham, MA, USA), 0.2% bovine serum albumin (BSA, Thermo Fisher Scientific), and 1.8 mM EGTA in Dulbecco’s Modified Eagle’s Medium/F12 (DMEM/F12, Thermo Fisher Scientific) for 15 min. The follicles were then punctured in DMEM/F12 containing 10% fetal bovine serum (FBS; Thermo Fisher Scientific) and 1% penicillin–streptomycin (Thermo Fisher Scientific) to obtain GCs.

### 2.6. Extraction and Quantitative PCR

Mice fed ND or HFD for 16 weeks (20 per group) were used for GC isolation. Total RNA was isolated from pooled samples consisting of GCs isolated from four mice (ND- or HFD-fed mice). Total RNA was extracted using TRIzol^®^ Reagent (Invitrogen, Carlsbad, CA, USA). Briefly, the tissues were homogenized in TRIzol (500 μL for the ovary and 300 μL for the pooled GCs), followed by the addition of one-fifth the volume of chloroform. After thorough mixing and incubation at 25 °C for 15 min, the samples were centrifuged at 4 °C for 20 min. The aqueous phase was transferred to a fresh tube, and one-tenth the volume of 3 M sodium acetate and an equal volume of isopropanol were added. After vortexing and centrifugation at 15,956× *g*, the resulting RNA pellet was washed twice with 80% ethanol, air-dried, and resuspended in 20 μL of nuclease-free water. The RNA was treated with DNase (Promega, Madison, WI, USA) for 20 min at room temperature to denature the DNA, and the DNase was inactivated by incubation at 55 °C for 10 min. The RNA concentration and quality were assessed using the spectrophotometer (NanoDrop^®^ One; Thermo Fisher Scientific, Waltham, MA, USA). Complementary DNA (cDNA) was synthesized from 2 μg of RNA using random hexamer primers (Invitrogen), oligo (dT) primers, M-MLV reverse transcriptase (Promega), and RNase inhibitor (Promega). To quantify the gene expression levels, reverse transcription-quantitative PCR (RT-qPCR) was performed with SYBR^®^ green dye (Bio-Rad Laboratories, Hercules, CA, USA) using 2 μL of cDNA in a final volume of 20 μL. A CFX Duet Real-Time PCR system (Bio-Rad Laboratories) was used for the analysis. Gene expression was normalized to that of the housekeeping gene *ribosomal protein L7* (*Rpl7*). The primer sequences used in this study are listed in Table 1. GraphPad Prism software (version 5; GraphPad Software Inc., La Jolla, CA, USA) was used to construct barograms and perform Student’s *t*-tests. One-way analysis of variance and Tukey’s honestly significant difference test were performed using ChatGPT-4 (OpenAI, San Francisco, CA, USA).

### 2.7. RNA-Sequencing

Mice fed ND or HFD for 16 weeks (20 per group) were used for GC isolation. Total RNA was isolated from pooled samples consisting of GCs isolated from four mice (ND- or HFD-fed mice). RNA sequencing was performed by LAS (Gimpo, Republic of Korea). RNA quality was assessed using the Agilent TapeStation 4000 System (Agilent Technologies, Amstelveen, the Netherlands). RNA was quantified using a ND-2000 spectrophotometer (Thermo Fisher Scientific). RNA libraries were constructed using a QuantSeq 3 mRNA Seq Library Prep Kit (Lexogen Inc., Vienna, Austria) according to the manufacturer’s instructions. High-throughput 75 single-end cycle sequencing was performed using NextSeq 550 (Illumina Inc., San Diego, CA, USA). The quality of the reads was examined using fast QC (http://www.bioinformatics.babraham.ac.uk/projects/fastqc/, accessed 19 December 2016), and low-quality (Phred quality score < 20) reads were trimmed using BBDuk, a part of bbmap software (http://sourceforge.net/projects/bbmap/, accessed 2 September 2019). After removing low-quality reads, the sequencing reads were aligned by Bowtie2 [27] using the reference genome of the Illumina iGenomes UCSC mm10. Reads were quantified using the genes.gtf gene annotation file from the Illumina iGenomes UCSC mm10 with BedTools [28]. After excluding 1199 microRNAs, 23,282 protein-coding mRNAs were analyzed.

From a total of 23,282 genes in the raw counts, 6852 low-expression genes (with no more than 10 reads in at least five samples) were excluded, leaving 16,430 genes for subsequent analyses. Differential expression (DE) analysis of the transcriptome data was conducted using DESeq2 1.44.0 [29]. For identifying DE genes (DEGs) between ND and HFD mice, we employed DESeq2 *p* < 0.01 and |log_2_ fold change| ≥ 0.5 as the criteria. A false discovery rate was applied to adjust the *p*-values. The top DEGs with >1.5-fold change (*p* < 0.05) are represented in the heatmap. Raw data for RNA-seq are deposited in the Korea Bio Data Station (https://kbds.re.kr, accessed on 3 December 2024, accession number KAP241039).

### 2.8. Statistical Analysis

For principal component analysis (PCA), the variance-stabilizing transformation in DESeq2 [29] was applied to the raw counts. PCA was performed using the R statistical package. For the hierarchical clustering analysis, we first normalized the raw counts according to the library size of each sample using the DESeq2 package and transformed them into a log2 scale. Pearson’s correlation coefficient was used to calculate the distance between samples, and a complete linkage option was chosen. A hierarchical clustering analysis was performed using the R statistics package. PCA and volcano plots were obtained using the gplot2 R package [30]. Heatmaps were drawn using the Complex Heatmap package in R. All data were analyzed using R version 4.3.0.

### 2.9. Western Blotting

For Western blotting, each sample was prepared from a single ovary. The collected ovary was homogenized in RIPA buffer [10 mM Tris (pH 7.2; HanLAB, Cheongju-si, Republic of Korea), 150 mM NaCl (Thermo Fisher Scientific), 0.1% Triton X-100 (Sigma-Aldrich, St. Louis, MO, USA), 5 mM EDTA (HanLAB), 1% SDS (Bio-Rad), 1 mM DTT (Sigma-Aldrich), 1 mM PMSF (MP Biomedicals, Irvine, CA, USA), and a protease inhibitor cocktail (Roche, Basel, Switzerland)]. After homogenization, the samples were centrifuged at 15,928× *g* for 20 min at 4 °C. Protein concentrations were measured using the BCA protein assay kit (Thermo Fisher Scientific). The extracted proteins were separated on a 10% SDS–polyacrylamide gel and subsequently transferred onto nitrocellulose membranes (Bio-Rad). Detection of chemiluminescent signals was performed using the WestFemto kit (Thermo Fisher Scientific). The protein expression levels were normalized to β-tubulin (TUBB) or glyceraldehyde 3-phosphate dehydrogenase (GAPDH) levels and quantified with NIH ImageJ software v1.54g. Data are presented as the mean ± standard error of the mean (SEM). All Western blotting experiments were repeated at least three times, and one representative set is shown. Details of the primary antibodies used are provided in Table 2.

### 2.10. Immunofluorescence Staining

The isolated GCs were seeded on glass coverslips and placed in a 12-well plate at a density of 2 × 10^5^ cells/coverslip. GCs were fixed in 4% paraformaldehyde (PFA) in phosphate-buffered saline (PBS; Gibco, Thermo Fisher Scientific) for 20 min and washed thrice with PBS for 5 min each. Cells were permeabilized with 0.25% Triton X-100 (Sigma-Aldrich) for 10 min. To prevent non-specific binding, the cells were treated with 2% BSA in PBS for 1 h at room temperature. The cells were then incubated with primary antibodies (Table 2) at 4 °C overnight. After washing, the cells were incubated with secondary antibodies for 1 h at room temperature in the dark. The DNA was counterstained with TO-PRO-3-iodide (Invitrogen). Coverslips were mounted on glass slides using the Vectashield mounting medium (Vector Laboratories, Newark, CA, USA) and observed under a Zeiss LMS900 confocal microscope (Carl Zeiss AG, Oberkochen, Germany). The images were obtained and analyzed using ZEN Blue software version 3.4 (Carl Zeiss AG).

Paraffin-embedded ovarian tissue sections were deparaffinized in xylene and rehydrated in serial dilutions of ethanol and PBS. The slides were subjected to antigen retrieval using an Antigen Unmasking Solution (Vector Laboratories). To obtain cryosections, sucrose-embedded ovaries were frozen, cut into 12 μm sections, and fixed with 4% PFA for 20 min. The cryosections were then treated with 0.25% Triton X-100 for 10 min and blocked with 2% BSA. The sections were then incubated with the primary antibody at 4 °C overnight. The following day, the sections were incubated with secondary antibodies at room temperature for 1 h in the dark. All experiments accompanied the negative control stained with the corresponding IgG as the primary antibody. The negative control experiments did not generate any signal. The antibodies used are listed in Table 2.

## 3. Results

### 3.1. Evaluation of the Physiological and Histological Alterations in ND and HFD Mice

To screen genes influenced by a HFD in GCs, we used a widely used model of diet-induced obesity in C57BL/6J mice [31,32,33,34]. Mice were randomly divided into two groups (25 mice per group), and each group was either fed ND or HFD for a specified period (Figure 1). As reported previously by investigations using similar models, HFD mice showed expected serological and histological changes (Figure 1). HFD mice showed a significant increase in body weight starting in the second week compared to ND mice. At the end of the experimental period, the HFD mice were approximately 70% heavier than the ND mice (Figure 1C). The fasting blood glucose levels (measured once a month) were significantly higher in HFD mice than those of ND mice at all measurement points (Figure 1C). By the 17th week, the HFD group demonstrated an approximately 34% increase in blood glucose levels compared to the ND group. At the experimental endpoint, the mice were fasted for 12 h, and blood samples were collected for lipid and glucose measurements. As shown in Figure 1D, significant increases in the levels of T-Chol, GLU, and HDL were confirmed in HFD mice, whereas the LDL and TG levels were not substantially different between the two groups. These results indicate that a prolonged HFD alters the physiological metabolic parameters, consistent with the results observed in other investigations [31,32,33,34]. As reported previously [31], the liver and visceral adipose tissue sections from HFD mice showed an increase in the volume of lipid droplets (Figure 1E, black arrows).

### 3.2. RNA-Sequencing (RNA-Seq) Analysis of GCs from ND and HFD Mice

**Figure 2:**
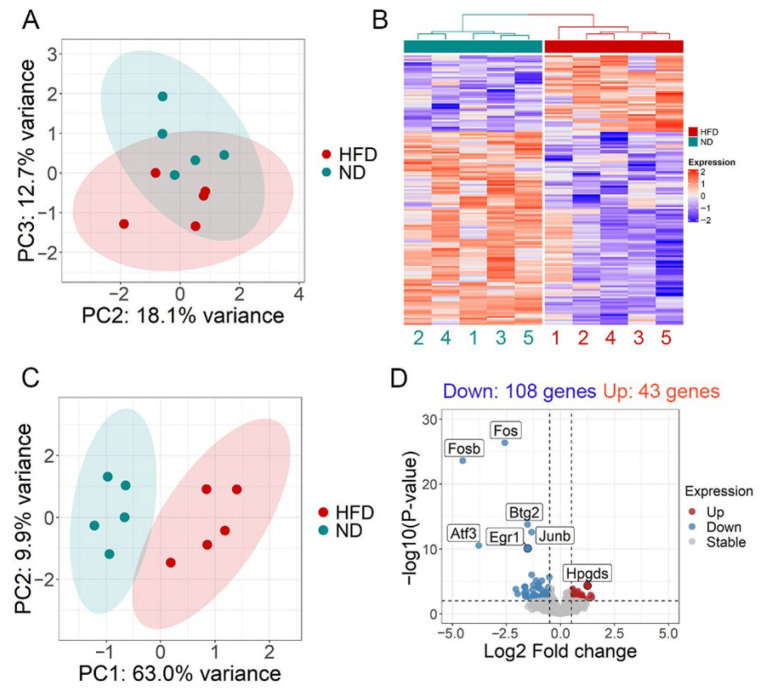
mRNA expression profiling of granulosa cells (GCs) isolated from ND and HFD ovaries. (**A**) Principal component analysis (PCA) plot illustrating the variance between ND and HFD along the second (PC2) and third (PC3) principal components. Based on PC3, HFD and ND appeared to show some separation. (**B**) A hierarchical clustering heatmap of differentially expressed genes (DEGs) in GCs from ND and HFD ovaries. Green and red bars on the top of the heatmap represent five sets of ND and HFD GCs, respectively. (**C**) PCA plot of DEGs between ND and HFD GCs showing that the HFD group is clearly separated from the ND group by the first PCA (PC1). (**D**) A volcano plot depicting representative DEGs between the ND and HFD groups is shown.

We performed RNA-seq analysis to investigate the transcriptomic changes in GCs isolated from ND and HFD mice. In the principal component analysis (PCA), the first two principal components (PC1 and PC2), which had the highest explanatory power for the transcriptome, were not significantly different between the ND and HFD groups (Figure S1). The HFD and ND groups were well separated, according to the third principal component (PC3), as shown in Figure 2A. To identify the genes associated with PC3, we performed a differentially expressed gene (DEG) analysis. A total of 151 DEGs were identified between the ND and HFD groups. Of these genes, 43 were upregulated and 108 were downregulated in HFD mice compared to ND mice (Figure 2B). A PCA plot based on these DEGs also clearly separated HFD mice and ND mice according to PC1 (Figure 2C). A few representative DEGs are shown in Figure 2D. Several gene ontology (GO) pathways were prominent among the downregulated DEGs. A list of DEGs and GO terms is provided in Table S1 (Supplementary Materials).

### 3.3. Downregulation of Immediate Early Transcription Factors in HFD GCs

**Figure 3:**
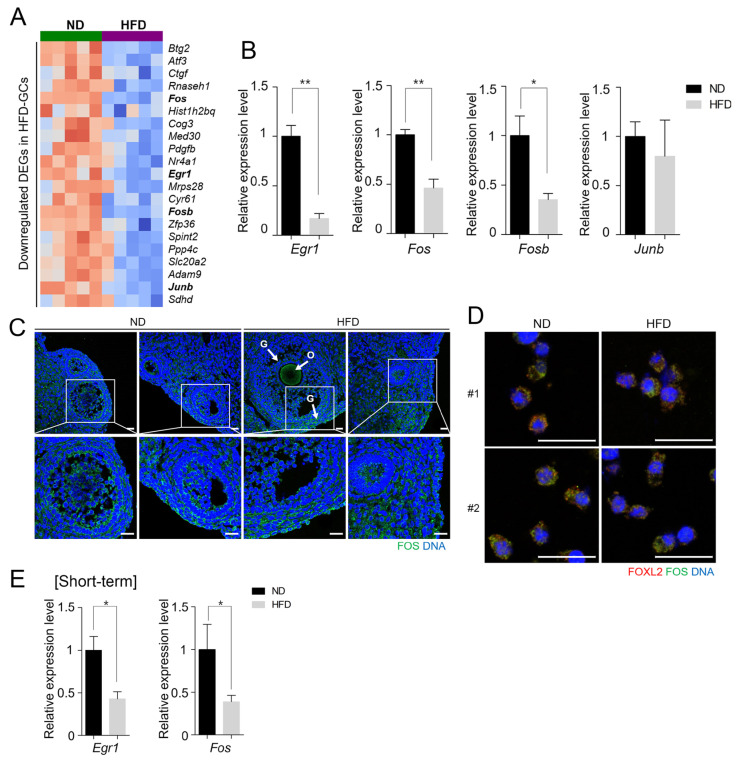
Downregulation of genes encoding immediate early transcription factors in granulosa cells (GCs) of HFD mice. (**A**) Partial heatmap of the downregulated DEGs in HFD GCs showing the top 21 downregulated genes. Red and blue represent the relative expression intensities (red, high; blue, low). Four genes encoding immediate early transcription factors are shown in bold. (**B**) RT-qPCR expression analyses of *Egr1*, *Fos*, *Fosb*, and *Junb* in GCs from ND and HFD mice. Each RNA sample was obtained from pooled GCs from four mice. Four independent sets of samples were used in duplicates. All values were normalized to *Rpl7* mRNA expression levels. Statistical analysis was performed using the *t*-test. * *p* < 0.05; ** *p* < 0.01. (**C**) Immunofluorescence staining of FOS in ovarian sections. Paraffin-embedded sections of ovaries from ND and HFD mice were treated with Antigen Unmasking Solution and subjected to immunofluorescence staining. Sections were probed with anti-FOS primary antibody and Alexa Fluor^®^ 488-conjugated chicken anti-rabbit IgG secondary antibody (green). DNA was counterstained with TO-PRO^TM^-3 iodine (blue). Two representative images are shown for each group. The areas in the white rectangles are enlarged in the bottom panel. The experiments were repeated three times. O, oocytes; G, granulosa cells. Scale bar = 20 µm. (**D**) Immunofluorescent staining of FOS and FOXL2 in isolated GCs. Two representative images are shown for each group (#1 and #2). GCs isolated from ND and HFD mice were co-stained with rabbit polyclonal anti-FOS (green) and goat polyclonal anti-FOXL2 antibodies, a GC marker (red). The cells were then probed with Alexa Fluor^®^ 568 donkey anti-rabbit IgG (1:250) and Alexa Fluor^®^ 488 chicken anti-goat IgG (1:250) secondary antibodies. DNA was counterstained with TO-PRO^TM^-3 iodine (blue). Two representative images are shown for each group. Scale bar = 20 µm. (**E**) Quantitative analyses of the Egr1 and Fos levels in GCs from short-term ND and HFD mice using RT-qPCR. Four independent sets of samples were assessed in duplicate. All values were normalized to Rpl7 mRNA expression levels. Statistical analyses were performed using the *t*-test. * *p* < 0.05.

A heatmap of the top 21 downregulated DEGs in the HFD GCs (Figure 3A) revealed the downregulation of several immediate early genes encoding transcription factors, including *activating transcription factor 3* (*Atf3*), *FBJ osteosarcoma oncogene* (*Fos*), *FBJ osteosarcoma oncogene B* (*Fosb*), and *Jun B proto-oncogene* (*Junb*) belonging to the activator protein-1 (AP-1) family of transcription factors that bind to specific regulatory regions to modulate gene expression [35]. *Early growth response 1* (*Egr1*), also an immediate early gene that contains a zinc-finger DNA-binding domain [36], is indispensable for ovulation in mice and is known to be induced by gonadotropins [37,38]. In the ovary, FOS acts as a critical downstream mediator of the progesterone receptor and epidermal growth factor signaling and increases the expression of genes important for ovulation [39]. Thus, *Egr1* and *Fos* were selected for further evaluation due to their involvement in ovarian functions [37,38,39].

We first performed RT-qPCR analysis using separate sample sets of GCs to confirm that the *Egr1*, *Fos*, and *Fosb* mRNA levels were significantly downregulated in the HFD GCs (Figure 3B). Immunofluorescence staining of FOS in ovarian sections and isolated GCs showed that it colocalized with FOXL2, a GC marker [40] (Figure 3C,D).

We next examined if an acute diet change, such as shorter-term HFD, similarly affects gene expression in the ovary. As the RNA-seq data were obtained from GCs of mice following 14–16 weeks of a HFD, we sought to investigate whether a similar change in gene expression occurs in GCs of mice with 4 weeks of a HFD (Figure 1B, four weeks of a HFD). As shown in Figure 3E, the expression of *Egr1* and *Fos* was downregulated, consistent with the results from the long-term HFD group, suggesting that HFD induces early changes in gene expression.

### 3.4. Regulation of Egr1 by Gonadotropins and the Effect of a HFD

**Figure 4:**
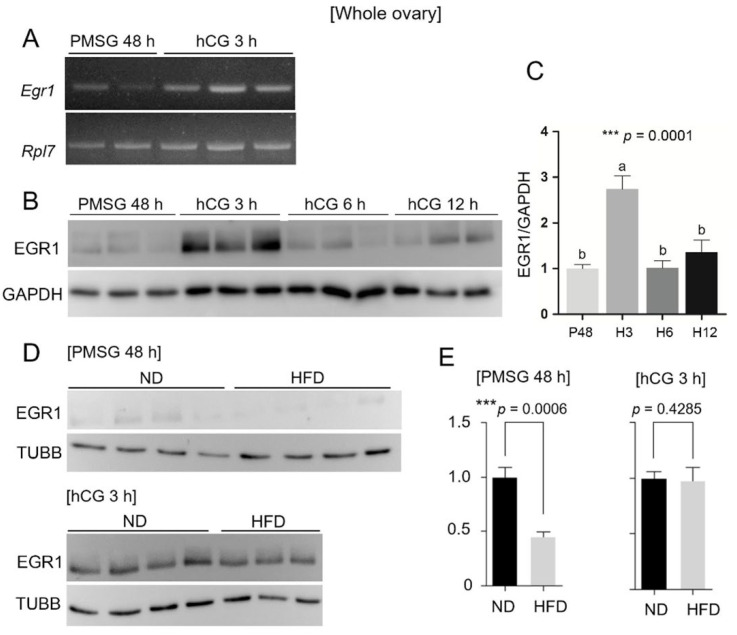
Differential expression of Egr1 in whole ovaries of gonadotropin-treated mice. (**A**) RT-PCR analysis of *Egr1* in whole ovaries of gonadotropin-treated mice at the indicated time points. All mice receiving hCG were primed with PMSG. PMSG 48 h (*n* = 2); hCG = 3 h (*n* = 3). (**B**) Western blotting of EGR1 in whole ovaries at the indicated time points. GAPDH, glyceraldehyde-3-phosphate dehydrogenase. As the TUBB levels fluctuated with different hormonal treatments, GAPDH was used as an internal control in this experiment. (**C**) The result in (**B**) was quantified using ImageJ software v1.54g and plotted as shown. The levels of TUBB were used to normalize the levels of EGR1. The mean and standard deviation (SD) for each experimental group (*n* = 3 for each group) were calculated. P48, PMSG 48 h; H3, hCG 3 h; H6, hCG 6 h; H12, hCG 12 h. A one-way analysis of variance (ANOVA) and Tukey’s test were performed using ChatGPT-4. Tukey’s post-hoc test found that H3 (a) differs significantly from other groups (b). *** *p* < 0.0001. (**D**) Western blotting of EGR1 in ND or HFD mice ovaries at the two indicated time points. (**E**) The results in (**D**) were quantified using ImageJ software v1.54g and plotted as shown. The levels of TUBB were used to normalize the levels of EGR1. The mean and standard deviation (SD) for each experimental group (*n* = 4 for each group) were calculated.

EGR1 is indispensable for ovulation in mice and is known to be induced by gonadotropins [37,38]. Specifically, EGR1 was previously shown to be induced at its highest level during the early hours of hCG induction [38]. We first confirmed this previous observation that the EGR1 levels fluctuated during gonadotropin administration, with the highest levels observed at 3 h post-hCG induction (Figure 4A,B). As our RNA-seq data were obtained from GCs at 48 h post-PMSG injection, we compared the expression of *Egr1* in HFD mice at two different time points: PMSG 48 h, at which our analysis was performed, and PMSG + hCG 3 h, at which *Egr1* showed the highest induction. The EGR1 levels were significantly lower in the HFD ovary at 48 h after PMSG injection, whereas, at 3 h, the levels were similar between the ND and HFD groups. These results confirmed that EGR1 was suppressed in HFD GCs at 48 h post-PMSG.

### 3.5. Upregulation of Inflammation-Associated Genes in HFD GCs

**Figure 5:**
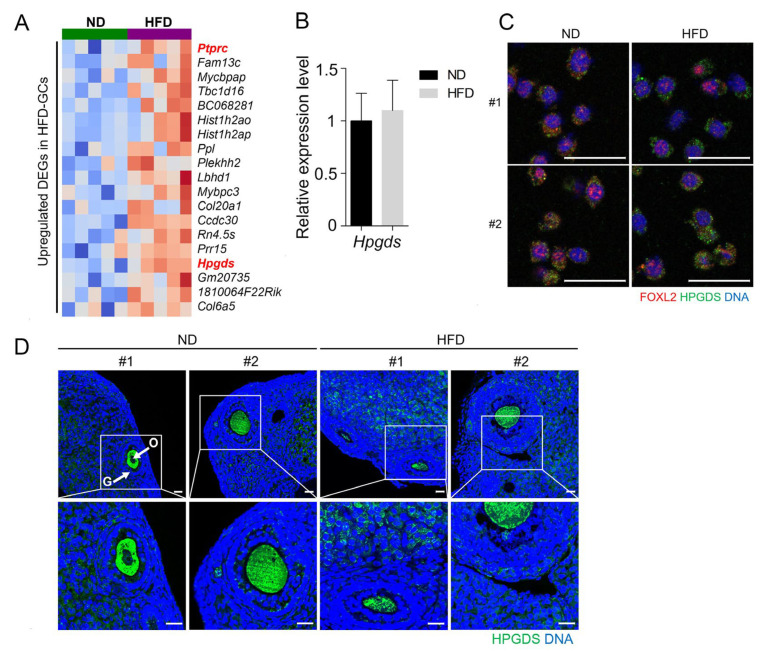
Upregulated genes in the granulosa cells (GCs) of HFD mice. (**A**) Partial heatmap of upregulated DEGs in HFD GCs. The top 19 upregulated DEGs are shown in the heatmap. Red and blue squares indicate high and low expression, respectively. The genes highlighted in red on the heatmap were selected for further evaluation. (**B**) Quantitative analysis *of hematopoietic type prostaglandin D synthase* (*Hpgds*) levels in GCs from ND and HFD mice by RT-qPCR. Four independent sets of samples were used in duplicates. All values were normalized to Rpl7 mRNA expression levels. Statistical analysis was performed using a *t*-test. (**C**) Immunofluorescence staining of HPGDS and FOXL2 in isolated GCs. GCs isolated from ND and HFD mice were co-stained with rat monoclonal anti-HPGDS (green) and goat polyclonal anti-FOXL2 antibodies (red), a GC marker. The cells were then probed with Alexa Fluor^®^ 488 donkey anti-rat IgG (1:250) and Alexa Fluor^®^ 546 rabbit anti-goat IgG (1:250) secondary antibodies. DNA was counterstained with TO-PRO^TM^-3 iodine (blue). Two representative images are shown for each group (#1 and #2). Scale bar = 20 µm. (**D**) Immunofluorescence staining of HPGDS in paraffin-embedded ovarian sections. Paraffin sections were treated with Antigen Unmasking Solution and subjected to immunofluorescence staining with an anti-HPGDS (green) primary antibody and Alexa Fluor^®^ 488 donkey anti-rat IgG secondary antibody. DNA was counterstained with TO-PRO^TM^-3 iodine (blue). The areas in the white rectangles are enlarged in the bottom panel. Two representative images are shown for each group (#1 and #2). The experiments were repeated three times. O, oocytes; G, granulosa cells. Scale bar = 20 µm.

Among the upregulated DEGs in HFD GCs (Figure 5A), *protein tyrosine phosphatase receptor type C* (*Ptprc*) and *hematopoietic prostaglandin D synthase* (*Hpgds*) were notable, as they are associated with increased inflammation. *Ptprc* encodes the cluster of differentiation 45 (CD45), a pan-hematopoietic cell marker and regulator of immune cell function [41], whereas HPGDS catalyzes the conversion of prostaglandin H_2_ into prostaglandin D_2_ (PGD_2_) in hematopoietic cells and other tissues [42,43]. As obesity is associated with increased inflammation in various tissues, including the ovary [44,45], the expression and localization of CD45 and HPGDS were further evaluated in HFD ovaries.

**Figure 6:**
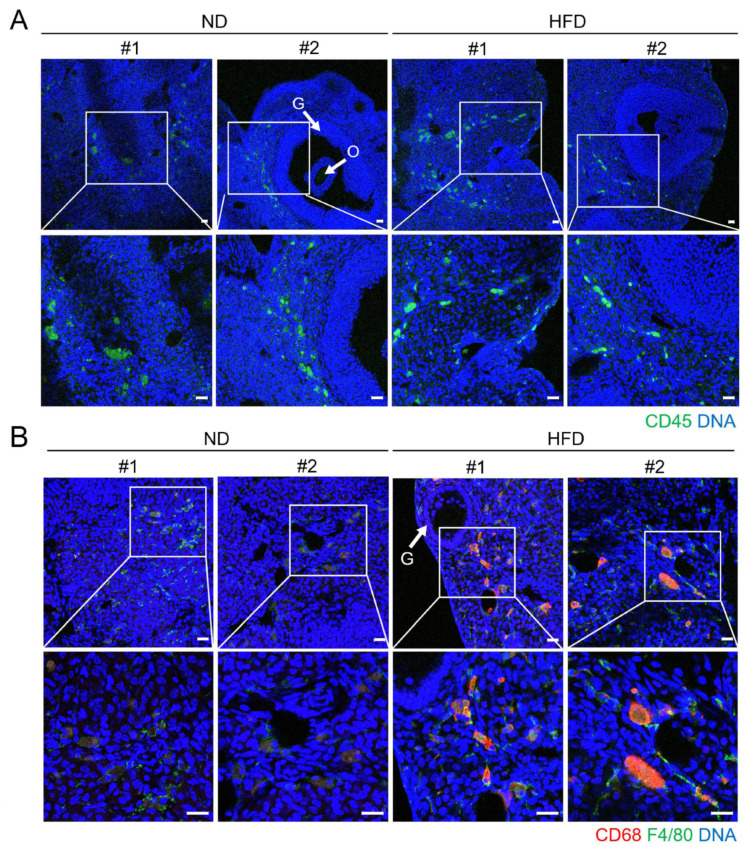
Localization of CD45-, CD68-, or F4/80-positive cells in ND and HFD mice ovaries. (**A**) Immunofluorescence staining of CD45 (encoded by *Ptprc*) in ovarian cryosections. A primary rat monoclonal anti-CD45 (green) antibody was used. The sections were then probed with Alexa Fluor^®^ 488 donkey anti-rat IgG secondary antibody. DNA was counterstained with TO-PRO^TM^-3 iodine (blue). Two representative images are shown for each group (#1 and #2), and the areas in the white rectangles are enlarged in the bottom panel. O, oocytes; G, granulosa cells. Scale bar = 20 µm. (**B**) Immunofluorescence staining of F4/80 and CD68 in ovarian cryosections. Primary rat monoclonal anti-F4/80 (green) and rabbit monoclonal anti-CD68 (red) antibodies were used. The sections were then probed with Alexa Fluor^®^ 488 donkey anti-rat IgG and Alexa Fluor^®^ 546 goat anti-rabbit IgG secondary antibodies. The DNA was counterstained with TO-PRO^TM^-3 iodine (blue). Two representative images are shown for each group (#1 and #2), and the areas in the white rectangles are enlarged in the bottom panel. G, granulosa cells. Scale bar = 20 µm.

As shown in Figure 5B, the *Hpgds* mRNA level was comparable in the GCs from ND and HFD mice. Immunofluorescence staining of the isolated GCs showed positive HPGDS signals, along with overlapping FOXL2, suggesting that these cells may produce PGD_2_ and influence the surrounding cellular environment (Figure 5C). In the ovarian cryosection, HPGDS was observed within the follicle, as well as in the stromal region in the HFD ovaries. Oocytes also showed positive signals (Figure 5D). Thus, HPGDS is localized both within and outside ovarian follicles. Since PGD_2_ produced by HPGDS is implicated in the increasing tissue infiltration of leukocytes, we used *Ptprc*-encoded CD45, a pan-leukocyte marker, to examine if there was an increased immune cell population within the HFD ovaries. As shown in Figure 6A, CD45-positive cells were present at various locations in both the ND and HFD ovaries.

### 3.6. Increased Infiltration of F4/80- and CD68-Positive Macrophages in HFD Ovaries

Among the upregulated DEGs, *Ptprc* and *Hpgds* have been implicated in increased inflammation [46,47,48,49]. As prolonged HFD may increase the infiltration of immune cells in the ovarian environment, we examined whether there was enhanced immune cell recruitment within the ovarian environment by evaluating F4/80- (a pan-macrophage marker) and CD68 (an activated-macrophage marker)-positive cells [50,51,52]. The combined use of F4/80 and CD68 markers is a common approach to identify and characterize macrophages in the ovarian tissue of mice [53]. Figure 6B shows that F4/80- and CD68-positive cells were distributed in the stromal regions of HFD ovaries.

## 4. Discussion

This study aimed to investigate the molecular and cellular changes in the ovarian environment of mice with diet-induced obesity. Various studies on the effects of diet on ovarian function have revealed its effects on oocyte quality, changes in follicular fluid, and pregnancy outcomes [54,55,56]. In this study, we examined the effects of a HFD on gene expression changes in isolated GCs of mice and identified significant alterations in the expression of several key genes involved in reproductive functions. As immediate early response transcription factors, EGR1 and members of the AP-1 family regulate early responses to growth stimuli and engage in a wide range of transcriptional regulatory processes associated with cellular proliferation, survival, inflammation, and differentiation [35,57,58]. As mentioned, FOS is known as a downstream mediator of the progesterone receptor and epidermal growth factor signaling in the ovary [39]. Thus, the downregulation of *Fos* implies that HFD may compromise ovulatory function in mice. EGR1 plays a crucial role in mediating luteinizing hormone responses necessary for steroidogenesis and ovulation [37,38] and the actions of steroid hormones in the uterus [59,60]. Thus, the reduced expression of these transcription factors in HFD GCs suggests that diet-induced metabolic stress may impair the ability of GCs to respond effectively to gonadotropins, potentially leading to the ovulatory dysfunction and subfertility observed in obesity. Among these factors, the association between *Egr1* and obesity has been documented in other systems, suggesting a certain level of complexity. In adipocytes, *Egr1* is induced by HFD and is linked to increased energy storage in white adipose tissue [61]. *Egr1* is induced in GCs isolated from obese pregnant rats during the preimplantation period [62]. As *Egr1*-deficient mice exhibit partial protection against fat deposition under HFD conditions [63], the possibility that EGR1 is a target of metabolic disorders is further strengthened by our findings. As for the human ovary, EGR1 is implicated in increasing the incidence of ovarian hyperstimulation syndrome [64], but whether ovarian EGR1 is associated with women’s body weight is unknown.

Obesity increases inflammation at a systemic level [65,66]. An increased infiltration of the immune cells has been reported in adipose tissue and the liver [67,68]. CD45, encoded by *Ptprc,* is expressed in almost all nucleated hematopoietic cells in mice [69] and is a key regulator of immune cell signaling [41]. CD45 has been implicated in the modulation of the local immune environment in the ovaries of patients with polycystic ovarian syndrome (PCOS) [48]. The significantly elevated *Ptprc* levels in HFD GC preparations in the present study may indicate the presence of more immune cells in HFD ovaries, accompanied by increased inflammation. Modulations in the immune factors in HFD ovaries may reflect an alteration in the inflammatory response or immune surveillance mechanisms within the ovarian microenvironment. Perturbation in the immunological environment can interfere with normal follicular development and ovulation. Similarly, the upregulation of *Hpgds*, an enzyme involved in prostaglandin D_2_ synthesis, suggests an increased production of PGD_2_, which, in turn, may enhance inflammatory or stress responses in the ovary. The expression of HPGDS has been reported in GCs [42] and in immune and mast cells during inflammatory responses [70]. Thus, the source of PGD_2_ that attracts immune cells may be the GCs or immune cells in the ovary.

The downregulation of *Egr1* and *Fos* was also observed in GCs from short-term HFD mice, which were under a HFD for 4 weeks. This observation is particularly important in the field of reproductive biology, as a prolonged period of special diet renders them older at the experimental endpoint, when their reproductive performance may be less robust. If a short-term diet change also produces similar effects, experimental designs become far less complex and can be performed with mice at younger ages. In this context, it is notable that one week of a HFD was reported to be sufficient to cause calcium signaling in the liver [71].

Our study provides novel insights into the early molecular changes in GCs induced by a HFD. We demonstrate, for the first time, that EGR1, a crucial transcription factor for ovulation, is significantly downregulated in response to a HFD in the ovary, even after short-term exposure. This suggests that metabolic stress may impair ovulatory function via EGR1 downregulation. Furthermore, we identify an increased expression of *Ptprc* and *Hpgds* in GCs, highlighting the implication of granulosa cells in modulating the ovarian inflammatory environment in obesity. Our findings also reveal that key transcriptional changes occur after only four weeks of HFD, underscoring the rapid impact of dietary composition on ovarian function and the potential for early intervention to mitigate obesity-associated reproductive dysfunction. Collectively, our findings highlight the complex interplay between the metabolic state, immune regulation, and reproductive function in GCs. Understanding the molecular changes in GCs in diet-induced obesity models could potentially lead to the development of targeted interventions or biomarkers for obesity-related ovarian malfunction. These basic findings may ultimately improve the diagnostic and treatment strategies for women struggling with obesity-associated infertility. Our results suggest that HFD-related metabolic dysregulation significantly alters the transcriptional landscape in GCs, potentially disrupting important pathways involved in ovarian function and fertility. Our results also provide insights into the mechanisms underlying the reduced efficacy of assisted-reproductive technologies in women with obesity. Further investigation is warranted to identify whether similar mechanisms operate in human GCs.

## Figures and Tables

**Table 1:** Primers used for the RT-qPCR analyses.

Gene	Primer Sequence (5’-3’)	Product Size (bp)	GenBank Accession No.
*Rpl7*	F: TCA ATG GAG TAA GCC CAA AG R: CAA GCG ACC GAG CAA TCA AG	246	NM_011291.5
*Egr1*	F: ACC CTA TGA GCA CCT GAC CAC R: TAT AGG TGA TGG GAG GCA ACC	116	NM_007913.5
*Fos*	F: GGT TTC AAC GCC GAC TAC GA R: GCG CAA AAG TCC TGT GTG TT	140	NM_010234.3
*Fosb*	F: ACC TGT CTT CGG TGG ACT CCT T R: TGG CTG GTT GTG ATT GCG GTG A	121	NM_001347586.1
*Junb*	F: GAC CTG CAC AAG ATG AAC CAC G R: ACT GCT GAG GTT GGT GTA GAC G	129	NM_008416.3
*Hpgds*	F: GGA GCA ATG TCA AGC TGA TGC AG R: TCA GAA GGC GAG GTG CTT GAT G	137	NM_019455.5

F, forward; R, reverse.

**Table 2:** Primary antibodies used in the study.

Antibody	Host	Cat. no	Supplier	Dilution
Anti-EGR1	Rabbit	4153	Cell Signaling	1:400
Anti-FOS	Rabbit	ab190289	Abcam	1:100
Anti-HPGDS	Rat	10004349	Cayman	1:200
Anti-CD45	Rat	NB100-77417SS	Novus Biologicals	1:200
Anti-CD68	Rabbit	ab283654	Abcam	1:100
Anti-F4/80	Rat	MCA497R	Bio-Rad	1:100
Anti-FOXL2	Goat	NB100-1277	Novus Biologicals	1:200
Anti-TUBB	Rabbit	ab6046	Abcam	1:1000
Anti-GAPDH	Mouse	6C5	Abcam	1:1000

## Data Availability

Raw data for RNA-seq are deposited in the Korea Bio Data Station (https://kbds.re.kr, accessed on 3 December 2024, accession number KAP241039). All data are fully accessible without restriction.

## Supplementary Materials

The following supporting information can be downloaded at https://www.mdpi.com/article/10.3390/life15030330/s1, Figure S1: Whole-transcriptome profiles of granulosa cells between normal diet (ND) and high-fat diet (HFD) mice. (A) A PCA plot illustrating the variance between the ND and HFD groups along PC1 and PC2. (B) A hierarchical clustering heatmap of all genes between the ND and HFD; Figure S2: The original images of the blots in Figure 3 and Figure 4; Table S1: The lists of differentially expressed genes (DEGs) and notable pathways.

## References

[1] Ferrulli A., Terruzzi I., Zuccotti G., Luzi L. (2023). Editorial for the Special Issue “Effects of COVID-19 on Lifestyle Behaviors in Children with Obesity”. Nutrients.

[2] Gambineri A., Laudisio D., Marocco C., Radellini S., Colao A., Savastano S., Obesity Programs of nutrition, Education, Research and Assessment (OPERA) Group (2019). Female infertility: Which role for obesity?. Int. J. Obes. Suppl..

[3] Gonnella F., Konstantinidou F., Di Berardino C., Capacchietti G., Peserico A., Russo V., Barboni B., Stuppia L., Gatta V. (2022). A Systematic Review of the Effects of High-Fat Diet Exposure on Oocyte and Follicular Quality: A Molecular Point of View. Int. J. Mol. Sci..

[4] Gonzalez M.B., Robker R.L., Rose R.D. (2022). Obesity and oocyte quality: Significant implications for ART and emerging mechanistic insights. Biol. Reprod..

[5] Minge C.E., Bennett B.D., Norman R.J., Robker R.L. (2008). Peroxisome proliferator-activated receptor-gamma agonist rosiglitazone reverses the adverse effects of diet-induced obesity on oocyte quality. Endocrinology.

[6] Jungheim E.S., Schoeller E.L., Marquard K.L., Louden E.D., Schaffer J.E., Moley K.H. (2010). Diet-induced obesity model: Abnormal oocytes and persistent growth abnormalities in the offspring. Endocrinology.

[7] Wu L.L., Dunning K.R., Yang X., Russell D.L., Lane M., Norman R.J., Robker R.L. (2010). High-fat diet causes lipotoxicity responses in cumulus-oocyte complexes and decreased fertilization rates. Endocrinology.

[8] Venkatesh S.S., Ferreira T., Benonisdottir S., Rahmioglu N., Becker C.M., Granne I., Zondervan K.T., Holmes M.V., Lindgren C.M., Wittemans L.B.L. (2022). Obesity and risk of female reproductive conditions: A Mendelian randomisation study. PLoS Med..

[9] Vahratian A., Smith Y.R. (2009). Should access to fertility-related services be conditional on body mass index?. Hum. Reprod..

[10] Collins S., Martin T.L., Surwit R.S., Robidoux J. (2004). Genetic vulnerability to diet-induced obesity in the C57BL/6J mouse: Physiological and molecular characteristics. Physiol. Behav..

[11] Speakman J., Hambly C., Mitchell S., Król E. (2007). Animal models of obesity. Obes. Rev..

[12] Wang C.Y., Liao J.K. (2012). A mouse model of diet-induced obesity and insulin resistance. Methods Mol. Biol..

[13] Sato A., Kawano H., Notsu T., Ohta M., Nakakuki M., Mizuguchi K., Itoh M., Suganami T., Ogawa Y. (2010). Antiobesity effect of eicosapentaenoic acid in high-fat/high-sucrose diet-induced obesity: Importance of hepatic lipogenesis. Diabetes.

[14] Han L., Ren C., Li L., Li X., Ge J., Wang H., Miao Y.L., Guo X., Moley K.H., Shu W. (2018). Embryonic defects induced by maternal obesity in mice derive from Stella insufficiency in oocytes. Nat. Genet..

[15] Montgomery M.K., De Nardo W., Watt M.J. (2019). Impact of Lipotoxicity on Tissue “Cross Talk” and Metabolic Regulation. Physiology.

[16] Lipke K., Kubis-Kubiak A., Piwowar A. (2022). Molecular Mechanism of Lipotoxicity as an Interesting Aspect in the Development of Pathological States-Current View of Knowledge. Cells.

[17] Luzzo K.M., Wang Q., Purcell S.H., Chi M., Jimenez P.T., Grindler N., Schedl T., Moley K.H. (2012). High fat diet induced developmental defects in the mouse: Oocyte meiotic aneuploidy and fetal growth retardation/brain defects. PLoS ONE.

[18] Reynolds K.A., Boudoures A.L., Chi M.M., Wang Q., Moley K.H. (2015). Adverse effects of obesity and/or high-fat diet on oocyte quality and metabolism are not reversible with resumption of regular diet in mice. Reprod. Fertil. Dev..

[19] Hohos N.M., Cho K.J., Swindle D.C., Skaznik-Wikiel M.E. (2018). High-fat diet exposure, regardless of induction of obesity, is associated with altered expression of genes critical to normal ovulatory function. Mol. Cell Endocrinol..

[20] Snider A.P., Wood J.R. (2019). Obesity induces ovarian inflammation and reduces oocyte quality. Reproduction.

[21] Zhu Q., Li F., Wang H., Wang X., Xiang Y., Ding H., Wu H., Xu C., Weng L., Cai J. (2024). Single-cell RNA sequencing reveals the effects of high-fat diet on oocyte and early embryo development in female mice. Reprod. Biol. Endocrinol..

[22] Edson M.A., Nagaraja A.K., Matzuk M.M. (2009). The mammalian ovary from genesis to revelation. Endocr. Rev..

[23] Richards J.S., Ascoli M. (2018). Endocrine, Paracrine, and Autocrine Signaling Pathways That Regulate Ovulation. Trends Endocrinol. Metab..

[24] Yao J., Li Z., Fu Y., Wu R., Wang Y., Liu C., Yang L., Zhang H. (2019). Involvement of obesity-associated upregulation of chemerin/chemokine-like receptor 1 in oxidative stress and apoptosis in ovaries and granulosa cells. Biochem. Biophys. Res. Commun..

[25] Hua D., Zhou Y., Lu Y., Zhao C., Qiu W., Chen J., Ju R. (2020). Lipotoxicity Impairs Granulosa Cell Function Through Activated Endoplasmic Reticulum Stress Pathway. Reprod. Sci..

[26] Campbell K.L. (1979). Ovarian granulosa cells isolated with EGTA and hypertonic sucrose: Cellular integrity and function. Biol. Reprod..

[27] Langmead B., Salzberg S.L. (2012). Fast gapped-read alignment with Bowtie 2. Nat. Methods.

[28] Quinlan A.R., Hall I.M. (2010). BEDTools: A flexible suite of utilities for comparing genomic features. Bioinformatics.

[29] Love M.I., Huber W., Anders S. (2014). Moderated estimation of fold change and dispersion for RNA-seq data with DESeq2. Genome Biol..

[30] Wickham H. (2016). Data Analysis. Ggplot2: Elegant Graphics for Data Analysis.

[31] Boudoures A.L., Chi M., Thompson A., Zhang W., Moley K.H. (2016). The effects of voluntary exercise on oocyte quality in a diet-induced obese murine model. Reproduction.

[32] Ota K., Komuro A., Amano H., Kanai A., Ge K., Ueda T., Okada H. (2019). High Fat Diet Triggers a Reduction in Body Fat Mass in Female Mice Deficient for Utx demethylase. Sci. Rep..

[33] Gao X., Li Y., Ma Z., Jing J., Zhang Z., Liu Y., Ding Z. (2021). Obesity induces morphological and functional changes in female reproductive system through increases in NF-κB and MAPK signaling in mice. Reprod. Biol. Endocrinol..

[34] Kim Y.N., Shin J.H., Kyeong D.S., Cho S.Y., Kim M.Y., Lim H.J., Jimenez M.R.R., Kim I.Y., Lee M.O., Bae Y.S. (2021). Ahnak deficiency attenuates high-fat diet-induced fatty liver in mice through FGF21 induction. Exp. Mol. Med..

[35] Shaulian E., Karin M. (2002). AP-1 as a regulator of cell life and death. Nat. Cell Biol..

[36] Swirnoff A.H., Milbrandt J. (1995). DNA-binding specificity of NGFI-A and related zinc finger transcription factors. Mol. Cell Biol..

[37] Lee S.L., Sadovsky Y., Swirnoff A.H., Polish J.A., Goda P., Gavrilina G., Milbrandt J. (1996). Luteinizing hormone deficiency and female infertility in mice lacking the transcription factor NGFI-A (Egr-1). Science.

[38] Russell D.L., Doyle K.M., Gonzales-Robayna I., Pipaon C., Richards J.S. (2003). Egr-1 induction in rat granulosa cells by follicle-stimulating hormone and luteinizing hormone: Combinatorial regulation by transcription factors cyclic adenosine 3′,5′-monophosphate regulatory element binding protein, serum response factor, sp1, and early growth response factor-1. Mol. Endocrinol..

[39] Choi Y., Rosewell K.L., Brännström M., Akin J.W., Curry T.E., Jo M. (2018). FOS, a Critical Downstream Mediator of PGR and EGF Signaling Necessary for Ovulatory Prostaglandins in the Human Ovary. J. Clin. Endocrinol. Metab..

[40] Schmidt D., Ovitt C.E., Anlag K., Fehsenfeld S., Gredsted L., Treier A.C., Treier M. (2004). The murine winged-helix transcription factor Foxl2 is required for granulosa cell differentiation and ovary maintenance. Development.

[41] Hermiston M.L., Xu Z., Weiss A. (2003). CD45: A critical regulator of signaling thresholds in immune cells. Annu. Rev. Immunol..

[42] Farhat A., Philibert P., Sultan C., Poulat F., Boizet-Bonhoure B. (2011). Hematopoietic-Prostaglandin D2 synthase through PGD2 production is involved in the adult ovarian physiology. J. Ovarian Res..

[43] Rossitto M., Ujjan S., Poulat F., Boizet-Bonhoure B. (2015). Multiple roles of the prostaglandin D2 signaling pathway in reproduction. Reproduction.

[44] Robker R.L., Wu L.L., Yang X. (2011). Inflammatory pathways linking obesity and ovarian dysfunction. J. Reprod. Immunol..

[45] Ellulu M.S., Patimah I., Khaza’ai H., Rahmat A., Abed Y. (2017). Obesity and inflammation: The linking mechanism and the complications. Arch. Med. Sci..

[46] Voskuhl R.R., Peterson R.S., Song B., Ao Y., Morales L.B., Tiwari-Woodruff S., Sofroniew M.V. (2009). Reactive astrocytes form scar-like perivascular barriers to leukocytes during adaptive immune inflammation of the CNS. J. Neurosci..

[47] Bridges P.J., Jeoung M., Shim S., Park J.Y., Lee J.E., Sapsford L.A., Trudgen K., Ko C., Gye M.C., Jo M. (2012). Hematopoetic prostaglandin D synthase: An ESR1-dependent oviductal epithelial cell synthase. Endocrinology.

[48] Adams J., Liu Z., Ren Y.A., Wun W.S., Zhou W., Kenigsberg S., Librach C., Valdes C., Gibbons W., Richards J. (2016). Enhanced Inflammatory Transcriptome in the Granulosa Cells of Women With Polycystic Ovarian Syndrome. J. Clin. Endocrinol. Metab..

[49] Rittchen S., Jandl K., Lanz I., Reiter B., Ferreirós N., Kratz D., Lindenmann J., Brcic L., Bärnthaler T., Atallah R. (2021). Monocytes and Macrophages Serve as Potent Prostaglandin D. Int. J. Mol. Sci..

[50] Wu R., Van der Hoek K.H., Ryan N.K., Norman R.J., Robker R.L. (2004). Macrophage contributions to ovarian function. Hum. Reprod. Update.

[51] Dahm-Kähler P., Ghahremani M., Lind A.K., Sundfeldt K., Brännström M. (2009). Monocyte chemotactic protein-1 (MCP-1), its receptor, and macrophages in the perifollicular stroma during the human ovulatory process. Fertil. Steril..

[52] Carlock C., Wu J., Zhou C., Ross A., Adams H., Lou Y. (2013). Ovarian phagocyte subsets and their distinct tissue distribution patterns. Reproduction.

[53] Turner E.C., Hughes J., Wilson H., Clay M., Mylonas K.J., Kipari T., Duncan W.C., Fraser H.M. (2011). Conditional ablation of macrophages disrupts ovarian vasculature. Reproduction.

[54] Hou Y.J., Zhu C.C., Duan X., Liu H.L., Wang Q., Sun S.C. (2016). Both diet and gene mutation induced obesity affect oocyte quality in mice. Sci. Rep..

[55] Ruebel M.L., Piccolo B.D., Mercer K.E., Pack L., Moutos D., Shankar K., Andres A. (2019). Obesity leads to distinct metabolomic signatures in follicular fluid of women undergoing in vitro fertilization. Am. J. Physiol. Endocrinol. Metab..

[56] Song J., Xiang S., Pang C., Guo J., Sun Z. (2020). Metabolomic alternations of follicular fluid of obese women undergoing in-vitro fertilization treatment. Sci. Rep..

[57] Bhattacharyya S., Fang F., Tourtellotte W., Varga J. (2013). Egr-1: New conductor for the tissue repair orchestra directs harmony (regeneration) or cacophony (fibrosis). J. Pathol..

[58] Khachigian L.M. (2021). Early Growth Response-1, an Integrative Sensor in Cardiovascular and Inflammatory Disease. J. Am. Heart Assoc..

[59] Kim H.R., Kim Y.S., Yoon J.A., Lyu S.W., Shin H., Lim H.J., Hong S.H., Lee D.R., Song H. (2014). Egr1 is rapidly and transiently induced by estrogen and bisphenol A via activation of nuclear estrogen receptor-dependent ERK1/2 pathway in the uterus. Reprod. Toxicol..

[60] Kim H.R., Kim Y.S., Yoon J.A., Yang S.C., Park M., Seol D.W., Lyu S.W., Jun J.H., Lim H.J., Lee D.R. (2018). Estrogen induces EGR1 to fine-tune its actions on uterine epithelium by controlling PR signaling for successful embryo implantation. FASEB J..

[61] Zhang J., Zhang Y., Sun T., Guo F., Huang S., Chandalia M., Abate N., Fan D., Xin H.B., Chen Y.E. (2013). Dietary obesity-induced Egr-1 in adipocytes facilitates energy storage via suppression of FOXC2. Sci. Rep..

[62] Ruebel M., Shankar K., Gaddy D., Lindsey F., Badger T., Andres A. (2016). Maternal obesity is associated with ovarian inflammation and upregulation of early growth response factor 1. Am. J. Physiol. Endocrinol. Metab..

[63] Bléher M., Meshko B., Cacciapuoti I., Gergondey R., Kovacs Y., Duprez D., L’Honoré A., Havis E. (2020). Egr1 loss-of-function promotes beige adipocyte differentiation and activation specifically in inguinal subcutaneous white adipose tissue. Sci. Rep..

[64] Wang H., Chen W., Huang Y., Sun Y., Liu Y., Zhu Y., Lu Z. (2023). EGR1 Promotes Ovarian Hyperstimulation Syndrome Through Upregulation of SOX9 Expression. Cell Transplant..

[65] Shoelson S.E., Herrero L., Naaz A. (2007). Obesity, inflammation, and insulin resistance. Gastroenterology.

[66] Hildebrandt X., Ibrahim M., Peltzer N. (2023). Cell death and inflammation during obesity: “Know my methods, WAT(son)”. Cell Death Differ..

[67] Liu R., Nikolajczyk B.S. (2019). Tissue Immune Cells Fuel Obesity-Associated Inflammation in Adipose Tissue and Beyond. Front. Immunol..

[68] Azzu V., Vacca M., Virtue S., Allison M., Vidal-Puig A. (2020). Adipose Tissue-Liver Cross Talk in the Control of Whole-Body Metabolism: Implications in Nonalcoholic Fatty Liver Disease. Gastroenterology.

[69] Dawes R., Petrova S., Liu Z., Wraith D., Beverley P.C., Tchilian E.Z. (2006). Combinations of CD45 isoforms are crucial for immune function and disease. J. Immunol..

[70] Kanaoka Y., Urade Y. (2003). Hematopoietic prostaglandin D synthase. Prostaglandins Leukot. Essent. Fat. Acids.

[71] Brumer R.P., Corrêa-Velloso J.C., Thomas S.J., Sandiford O.A., Thomas A.P., Bartlett P.J. (2023). Short-term high-fat diet feeding of mice suppresses catecholamine-stimulated Ca. J. Physiol..

