# Supplementary figures and images for: Diet-Induced Obesity Alters Granulosa Cell Transcriptome and Ovarian Immune Environment in Mice
Source: Life (Basel). 2025 Feb 20;15(3):330. doi: 10.3390/life15030330 (PMC11943477; doi:10.3390/life15030330)

A

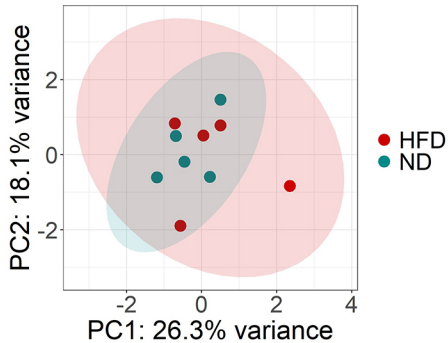

B

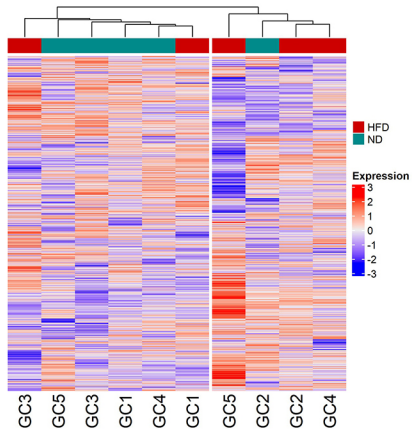

Supplement: Supplementary file 1 [file life-15-00330-s001.zip › Figure S1.pdf]

## Full uncropped images of gels and blots in Figure 4

(A)

*Rpl7* 28 cycle (246 bp)

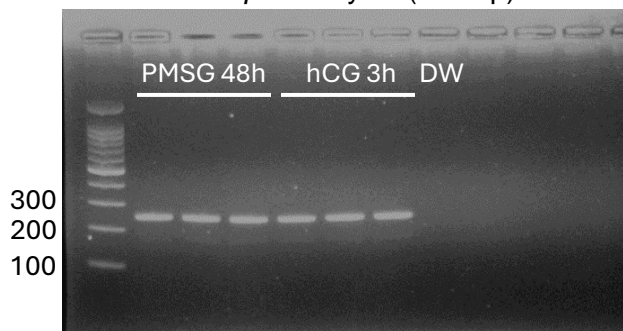

*Egr1* 33 cycle (116 bp)

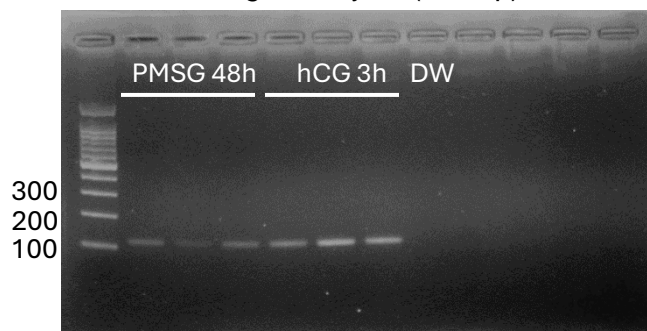

(B)

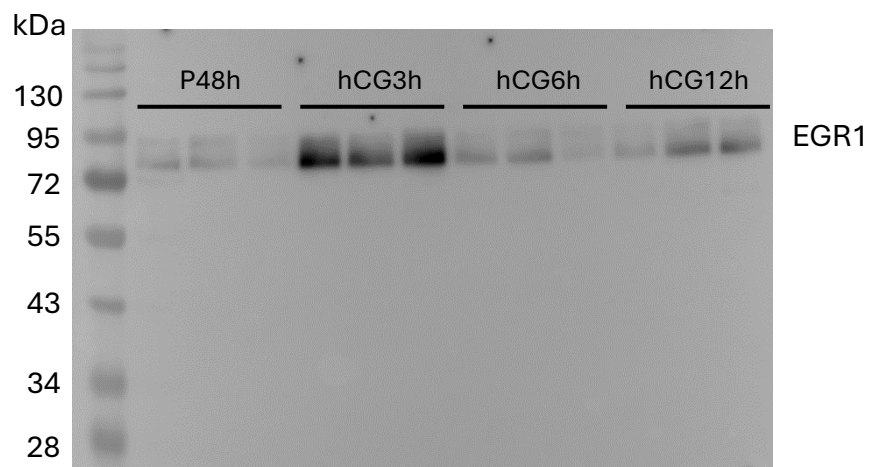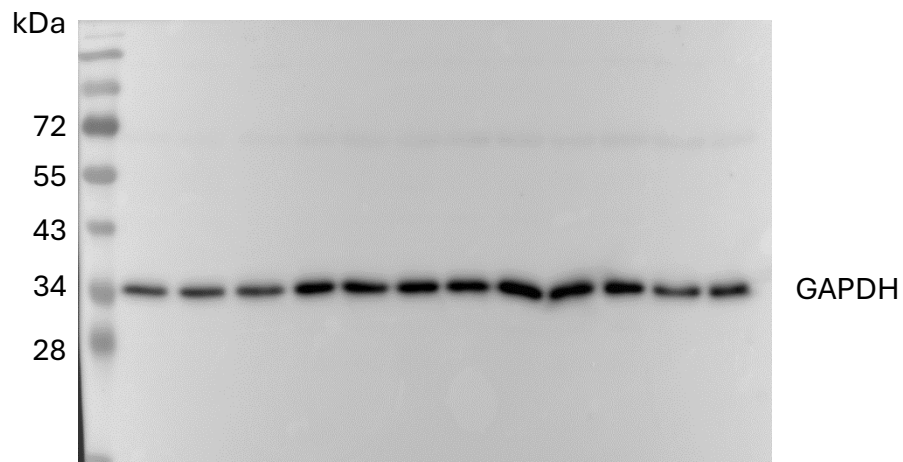

(D)

[PMSG 48h]

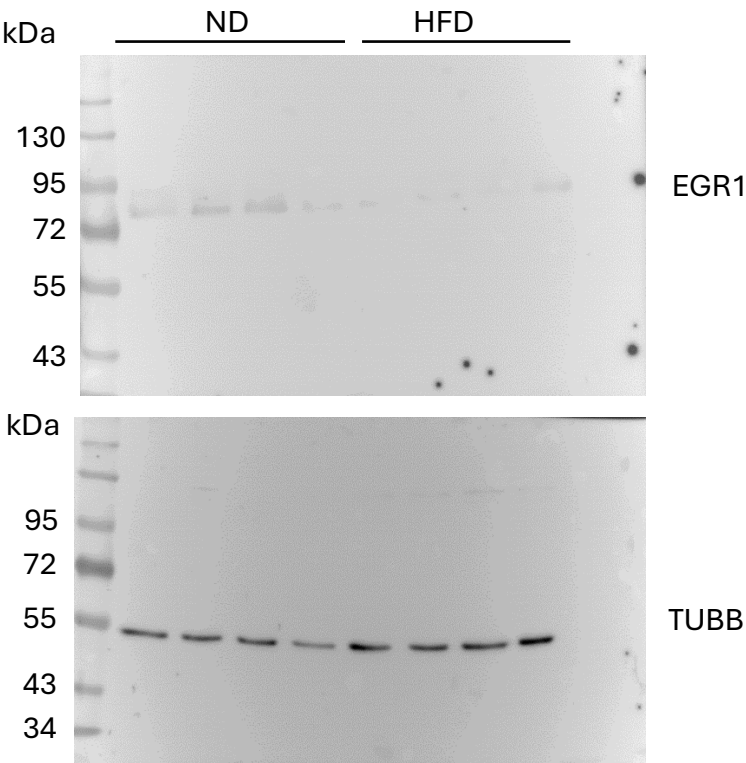

[hCG 3h]

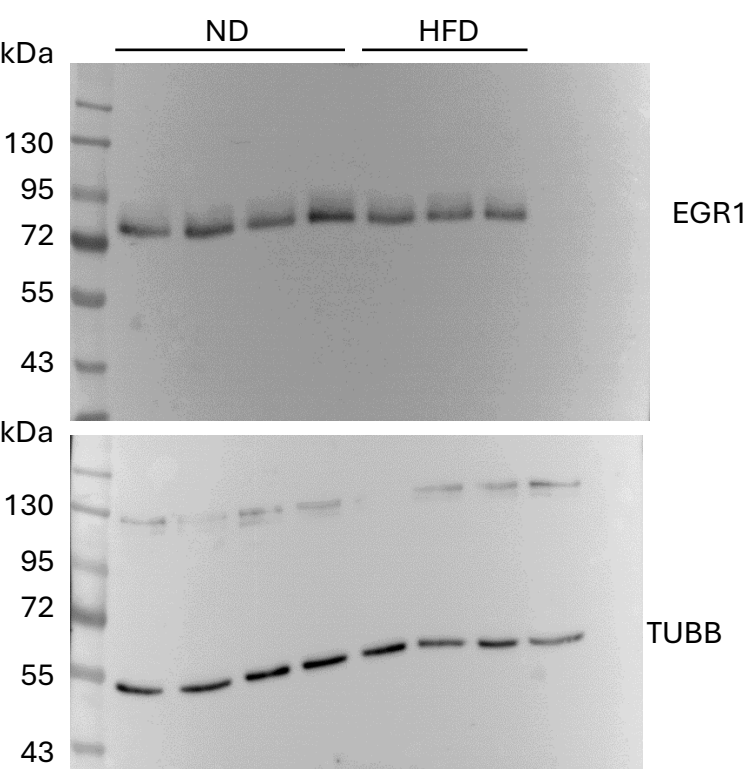

Supplement: Supplementary file 1 [file life-15-00330-s001.zip › Figure S2 Raw images_revised.pdf]
